# Skin Telocyte Secretome as Conditioned Medium Prevents Profibrotic Differentiation of Skin Fibroblasts into Myofibroblasts

**DOI:** 10.3390/ijms26031284

**Published:** 2025-02-02

**Authors:** Irene Rosa, Bianca Saveria Fioretto, Elena Andreucci, Alessio Biagioni, Eloisa Romano, Mirko Manetti

**Affiliations:** 1 Section of Anatomy and Histology, Department of Experimental and Clinical Medicine, University of Florence, Largo Brambilla 3, 50134 Florence, Italy; irene.rosa@unifi.it (I.R.); biancasaveria.fioretto@unifi.it (B.S.F.); 2 Imaging Platform, Department of Experimental and Clinical Medicine, University of Florence, Largo Brambilla 3, 50134 Florence, Italy; 3 Section of Experimental Pathology and Oncology, Department of Experimental and Clinical Biomedical Sciences “Mario Serio”, University of Florence, Viale Morgagni 50, 50134 Florence, Italy; e.andreucci@unifi.it (E.A.); alessio.biagioni@unifi.it (A.B.); 4 Section of Internal Medicine, Department of Experimental and Clinical Medicine, University of Florence, Largo Brambilla 3, 50134 Florence, Italy; eloisa.romano@unifi.it

**Keywords:** telocytes, secretome, fibroblasts, myofibroblasts, skin fibrosis, fibroblast-to-myofibroblast transition, TGFβ1

## Abstract

Telocytes (TCs) are distinctive cells widely localized in the stromal compartment of several human organs, including the skin. By means of their peculiar prolongations named telopodes, skin TCs are organized in networks interconnected with a variety of adjacent cells, being thus supposed to take part in skin homeostasis through both cell-to-cell contacts and the release of extracellular vesicles. A disarrangement/loss of the TC network was shown in human fibrotic skin as well as in the murine model of bleomycin-induced cutaneous fibrosis, but whether such TC alterations may represent just a consequence or a trigger of the fibrotic process still remains to be clarified. Thus, we investigated the effects of skin TC secretome as conditioned medium (TC-CM) on the transition of skin fibroblasts into myofibroblasts promoted by the master profibrotic cytokine transforming growth factor β1 (TGFβ1). Primary cultures of both adult human skin TCs and fibroblasts were obtained by means of immunomagnetic cell separation. Nanoparticle tracking analysis was carried out to measure extracellular vesicles in TC-CM. The combination of multiple morphological, gene/protein expression, and functional assessments demonstrated that TC-CM was able to significantly prevent TGFβ1-induced fibroblast-to-myofibroblast transition. TC-CM did not influence cell viability, while it effectively inhibited TGFβ1-induced fibroblast proliferation, migration, and morphological changes. Indeed, TC-CM was able to reduce TGFβ1-mediated skin fibroblast phenotypic and functional differentiation into myofibroblasts, as shown by a significant decrease in *FAP*, *ACTA2*, *COL1A1*, *COL1A2*, *FN1*, and *CTGF* gene expression, α-smooth muscle actin, N-cadherin, COL1A1, and FN-EDA protein levels, and collagen gel matrix contraction. Furthermore, TC-CM significantly lowered TGFβ1-mediated ERK1/2 signaling pathway activation. This in vitro study proves for the first time that TCs may play an important role in skin homeostasis through the prevention of fibroblast-to-myofibroblast transition via paracrine mechanisms and affords the necessary basis to investigate in the future the feasibility of TC secretome as an innovative antifibrotic therapeutic tool.

## 1. Introduction

Telocytes (TCs) are a peculiar cell population filling the stromal compartment of the skin and many other organs [1,2,3,4,5,6]. As demonstrated by several studies published over the last fifteen years, TCs are cells of mesenchymal origin that differ from “classical” fibroblasts in terms of their morphological and immunohistochemical profiles [2,5,7,8,9,10]. Indeed, TCs are easily recognizable by their small piriform, spindle or triangular cell body giving rise to their extremely long cytoplasmic prolongations named telopodes, which are characterized by a typical moniliform aspect due to the repetition of very slim segments (podomers) and small enlarged portions (podoms) [1,2,9]. In addition, at the ultrastructural level, many different types of extracellular vesicles (i.e., exosomes, ectosomes, and multivesicular cargos) can be observed around telopodes, suggesting a TC paracrine activity able to functionally regulate neighboring cells [11,12,13]. By light microscopy, from an immunohistochemical point of view, TC-specific markers have not yet been discovered and their antigenic profiles can differ according to the different tissues/organs [2,4,6,14]. However, since TCs were proved to express CD34 in almost every structure in which they have been identified, they are also defined as TCs/CD34+ stromal cells [5,6,8,15].

Spatially, TCs are usually organized into a three-dimensional labyrinth-like interstitial network that in normal skin compartmentalizes the dermis, with telopodes closely surrounding microvessels, nerve endings, and skin adnexa, as well as establishing intercellular contacts with several cell types including fibroblasts, mast cells, macrophages, and stem cells [7,13,16,17,18,19,20,21,22]. On the basis of (i) their characteristic spatial organization, (ii) the numerosity of cell-to-cell communications they establish, and (iii) their great ability to release extracellular vesicles, TCs are supposed to play a crucial role in the maintenance of local tissue homeostasis, the disruption of which may lead to different pathologic conditions affecting a variety of organs including the skin [2,4,6,15,20,23,24,25,26,27,28,29]. Indeed, TC alterations have been described in a wide range of skin diseases including cancer, chronic inflammatory conditions, and fibrotic disorders such as scleroderma, a complex connective tissue disease characterized by immune system disturbances, microvascular damage, and progressive skin fibrosis [19,30,31,32,33,34]. In particular, a disruption of the dermal network of TCs was reported not only in the fibrotic skin lesions of scleroderma patients, where it was correlated with the degree of fibrosis, but also in a widely used experimental murine model in which subcutaneous bleomycin injections lead to the development of scleroderma-like dermal fibrotic features [35,36]. Nevertheless, whether TC damage/disappearance may represent a mere consequence of the fibrotic process or could instead play an active role in the onset of skin fibrogenesis and in the progression of fibrotic lesions remains to be clarified.

Hence, in order to better unravel the putative contribution of TCs to cutaneous fibrosis, the current in vitro study investigated the effects of skin TC secretome as conditioned medium (TC-CM) on the transition of skin fibroblasts into myofibroblasts promoted by the master profibrotic cytokine transforming growth factor β1 (TGFβ1).

## 2. Results

### 2.1. Primary Human Skin Telocytes Release Extracellular Vesicles in Culture

**Figure 1:**
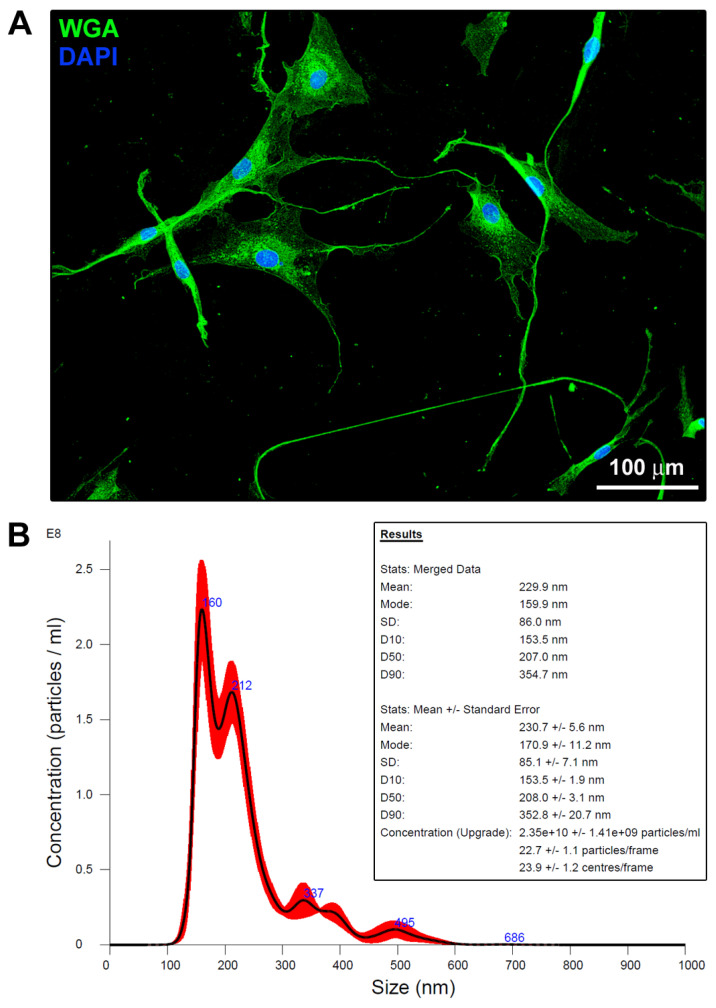
Primary human skin telocytes display distinctive morphological features and release extracellular vesicles. (**A**) Demonstrative fluorescence image of skin telocytes stained with fluorescent wheat germ agglutinin (WGA) that labels the plasma membrane. Telocytes display a variously shaped small cell body and typical very long and slim cytoplasmic extensions with a moniliform shape termed telopodes. Nuclei are counterstained blue with 4′,6-diamidino-2-phenylindole (DAPI). Scale bar: 100 µm. (**B**) Extracellular vesicle measurements in the skin telocyte conditioned medium. Demonstrative graph of NanoSight nanoparticle tracking analysis. Mode is the particle size value (nm) that corresponds to the peak of the distribution. D10, D50, and D90 are the sizes (nm) below which 10%, 50%, and 90% of all particles are found, respectively.

Primary human skin TCs were isolated by a previously established two-step immunomagnetic microbead-based cell separation process, as described elsewhere [8]. The purified skin TCs exhibited a small cell body and characteristic very long and slim telopodes (Figure 1A).

In order to verify the actual release of extracellular vesicles by skin TCs, we performed a nanoparticle tracking analysis of their conditioned medium, which highlighted the presence of several particles with different dimensions. As revealed by their distribution and concentration, the mean particle size we found was 230.7 ± 5.6 nm, while the mode, that is the particle size value (nm) corresponding to the peak of the distribution, was 170.9 ± 11.2 nm. In addition, the size below which 90% of all particles were found (D90) was 352.8 ± 20.7 nm. Taken together, these findings prompted us to suppose that the majority of TC-released extracellular vesicles can be assigned to the category of “classical” microvesicles ranging from 150 to 1000 nm (i.e., small to large ectosomes) [37].

### 2.2. Skin Telocyte Conditioned Medium Does Not Influence the Viability of Skin Fibroblasts but Significantly Prevents TGFβ1 Proliferative Effects

As previously shown in detail [8], the methodology used for TC isolation allowed for establishing, in parallel, primary cell cultures of both human skin TCs and fibroblasts from the same tissue donors (*n* = 3).

**Figure 2:**
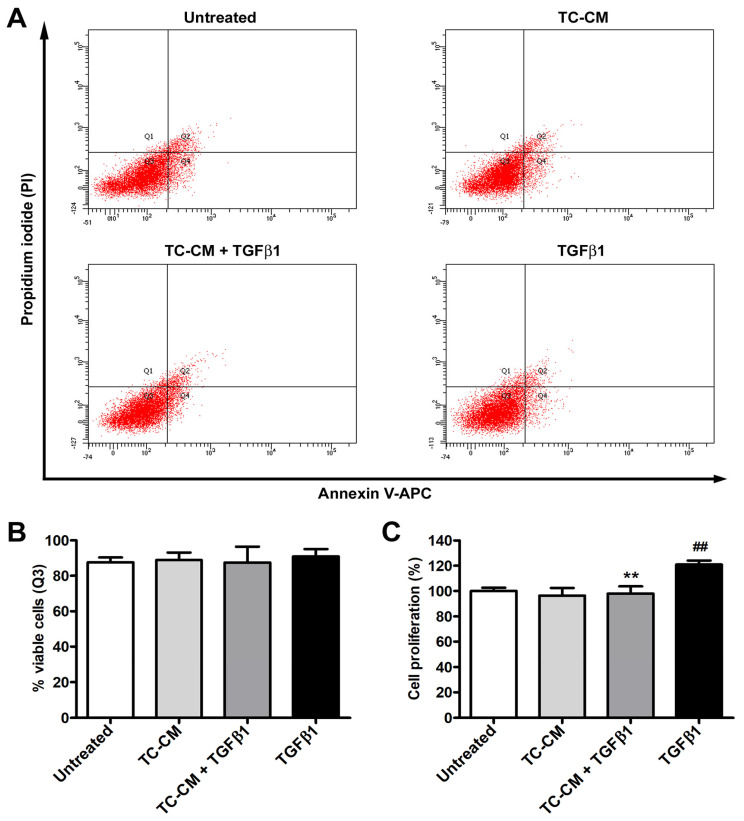
Skin telocyte conditioned medium (TC-CM) does not alter skin fibroblast viability but significantly prevents the proliferative effects of transforming growth factor β1 (TGFβ1). (**A**) Demonstrative annexin V/propidium iodide (PI) flow cytometry scatter plots for each experimental condition. (**B**) Percentage of annexin V−/PI− viable fibroblasts (Q3 quadrant in the flow cytometry scatter plots) for every experimental condition. (**C**) Fibroblast proliferation measured by WST-1 colorimetric assay. Proliferation of untreated fibroblasts was considered as 100% for normalization of results of the other experimental conditions. Values are mean ± SD of three independent experiments (*n* = 3 technical replicates for each experimental point) performed with three different cell lines of adult human skin telocytes and fibroblasts. ## *p* < 0.01 vs. untreated, ** *p* < 0.01 vs. TGFβ1 (Tukey’s test).

To verify whether TC-CM could potentially influence the viability of skin fibroblasts, we performed the annexin V/propidium iodide (PI) flow cytometry assay, and we observed no substantial modifications in the amount of viable, early/late apoptotic, and necrotic cells among the diverse experimental conditions (Figure 2A,B). As far as cell proliferation, WST-1 colorimetric assay not only confirmed that treatment with TGFβ1 alone was able to induce a strong increase in the proliferative rate of fibroblasts, but also showed that TC-CM had the ability to significantly dampen this effect (Figure 2C).

### 2.3. Skin Telocyte Conditioned Medium Significantly Reduces TGFβ1-Induced Fibroblast Ability to Close a Scratched Cellular Monolayer

**Figure 3:**
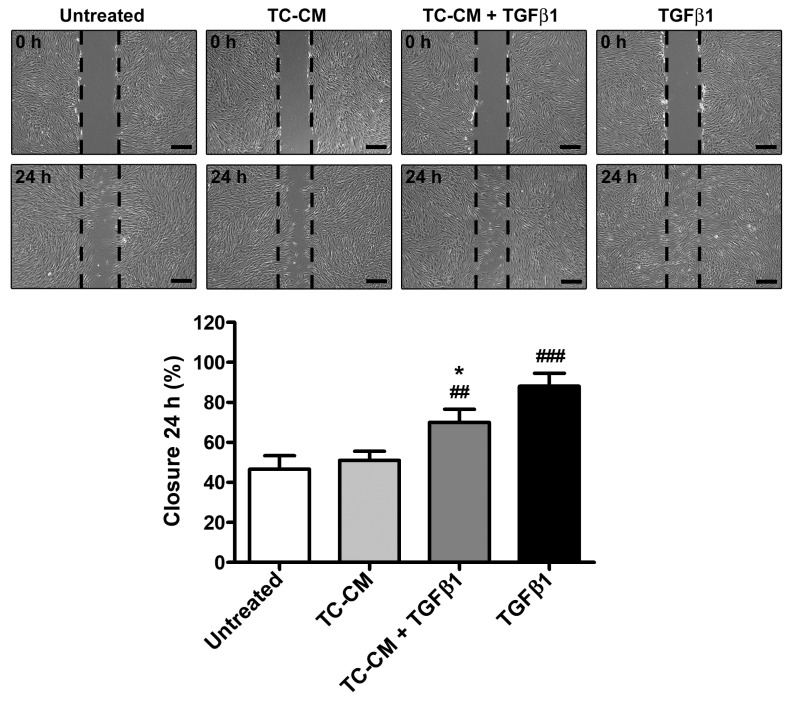
Skin telocyte conditioned medium (TC-CM) significantly reduces the transforming growth factor β1 (TGFβ1)-mediated ability of skin fibroblasts to restore the integrity of a scratched cellular monolayer. Demonstrative phase-contrast images of the culture plate scratched area at 0 and 24 h. Scale bar: 400 µm. The dashed black lines indicate the borders of the culture plate scratched area. Results are expressed as the percentage of closure of the scratched area at 24 h. Values are mean ± SD of three independent experiments (*n* = 3 technical replicates for each experimental point) performed with three different cell lines of adult human skin telocytes and fibroblasts. ### *p* < 0.001 and ## *p* < 0.01 vs. untreated, * *p* < 0.05 vs. TGFβ1 (Tukey’s test).

Human skin fibroblasts challenged with TGFβ1 exhibited a significant raise in their migratory ability, as demonstrated by a ~90% scratched area closure after 24 h (Figure 3). The administration of TC-CM was able to significantly reduce the TGFβ1-mediated effects, dropping the scratched area closure percentage up to ~70% (Figure 3).

### 2.4. Skin Telocyte Conditioned Medium Prevents Skin Fibroblasts to Acquire a TGFβ1-Induced Myofibroblast Morphology and Profibrotic Phenotype

**Figure 4:**
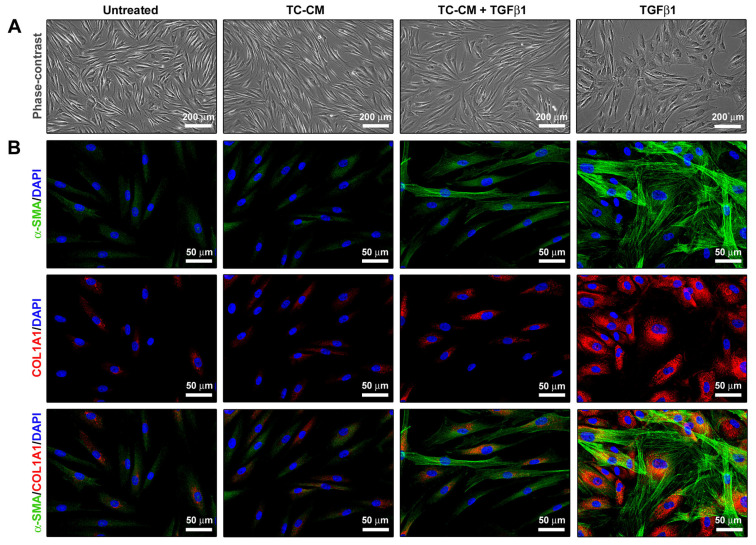
Skin telocyte conditioned medium (TC-CM) prevents the transforming growth factor β1 (TGFβ1)-induced gaining of myofibroblast morphology and profibrotic phenotype by skin fibroblasts. (**A**) Demonstrative phase-contrast images of skin fibroblasts for each experimental condition. (**B**) Demonstrative fluorescence images of skin fibroblasts immunolabeled for both α-smooth muscle actin (α-SMA) and α-1 chain of type I collagen (COL1A1) and counterstained with the 4′,6-diamidino-2-phenylindole (DAPI) nuclear staining dye. Scale bars: 200 µm (**A**), 50 µm (**B**).

As expected, treatment with TGFβ1 induced changes in normal skin fibroblast morphology toward a myofibroblast-like one, making them gain larger dimensions and a flattened and polygonal-shaped cell body (Figure 4A,B). Furthermore, when stimulated with TGFβ1, cells underwent a significant restructuring of the F-actin cytoskeleton, presenting with a larger amount of stress fibers (Figure 4A,B). Notably, TC-CM was able to lessen both TGFβ1-induced morphological and cytoskeletal variations (Figure 4A,B). Fluorescence immunocytochemistry confirmed that TGFβ1 stimulation significantly upregulated α-1 chain of type I collagen (COL1A1) and α-smooth muscle actin (α-SMA), with the latter highly organized into stress fibers (Figure 4B). As illustrated in Figure 4B, TC-CM strongly attenuated such an effect.

### 2.5. Skin Telocyte Conditioned Medium Reduces TGFβ1-Induced Skin Fibroblast Acquisition of Myofibroblast Markers and Contractile Ability

**Figure 5:**
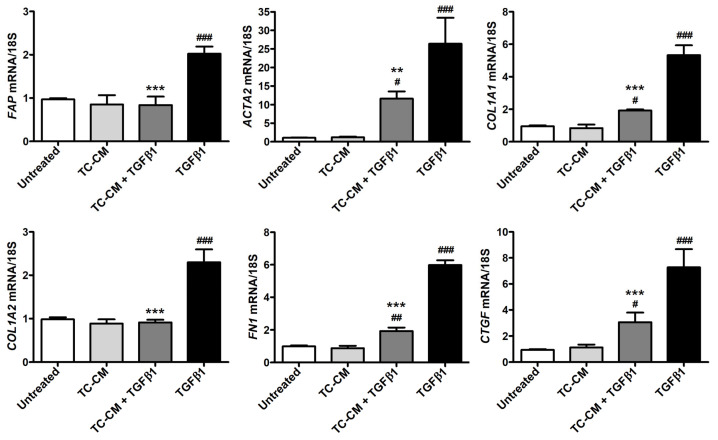
Skin telocyte conditioned medium (TC-CM) significantly dampens the transforming growth factor β1 (TGFβ1)-induced raise in gene expression of markers of myofibroblast differentiation and fibrogenesis in skin fibroblasts. *FAP*, *ACTA2*, *COL1A1*, *COL1A2*, *FN1*, and *CTGF* mRNA levels measured by quantitative real-time PCR in parallel with the 18S ribosomal RNA housekeeping gene are expressed as fold change with respect to those in untreated cells (set to 1). Values are mean ± SD of three independent experiments (*n* = 3 technical replicates for each experimental point) performed with three different cell lines of adult human skin telocytes and fibroblasts. ### *p* < 0.001, ## *p* < 0.01, and # *p* < 0.05 vs. untreated, *** *p* < 0.001 and ** *p* < 0.01 vs. TGFβ1 (Tukey’s test). *FAP*—gene encoding for fibroblast activation protein; *ACTA2*—gene encoding for α-smooth muscle actin; *COL1A1*—gene encoding for α-1 chain of type I collagen; *COL1A2*—gene encoding for α-2 chain of type I collagen; *FN1*—gene encoding for fibronectin 1; *CTGF*—gene encoding for connective tissue growth factor.

When performed on skin fibroblasts treated with TGFβ1 alone, quantitative real-time PCR showed a significant increase in *FAP*, *ACTA2*, *COL1A1*, *COL1A2*, *FN1* and *CTGF* gene expression (Figure 5). When in combination with TGFβ1, TC-CM was capable of significantly reducing the transcript levels of all these genes (Figure 5).

**Figure 6:**
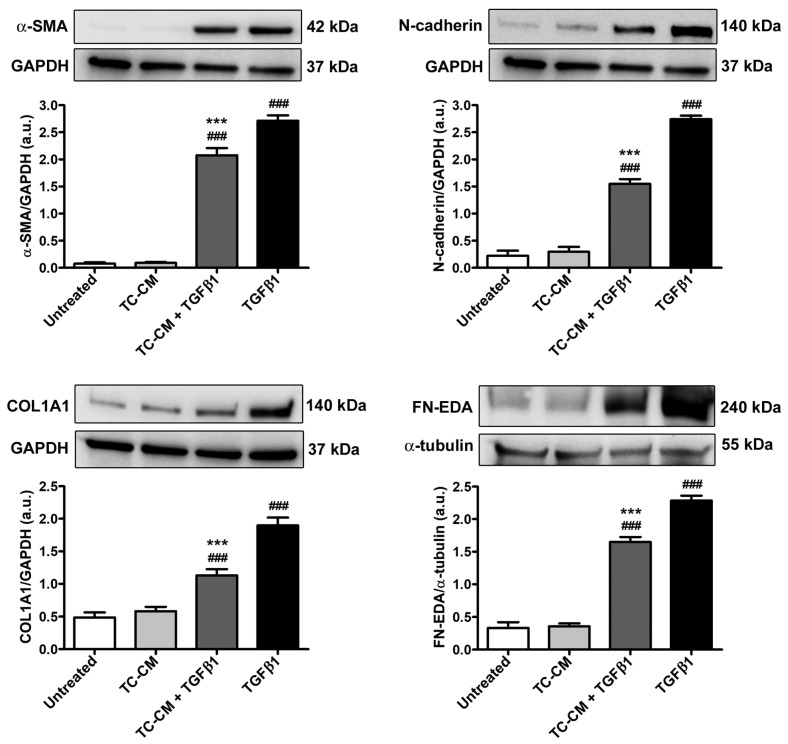
Skin telocyte conditioned medium (TC-CM) significantly dampens the transforming growth factor β1 (TGFβ1)-induced raise in protein levels of markers of myofibroblast differentiation and fibrogenesis in skin fibroblasts. Demonstrative bands of immunoblots for α-smooth muscle actin (α-SMA), N-cadherin, α-1 chain of type I collagen (COL1A1), and fibronectin containing the alternatively spliced extra domain A (FN-EDA). Glyceraldehyde 3-phosphate dehydrogenase (GAPDH) or α-tubulin measured on the same membrane serves as loading control for normalization. The molecular weight (kDa) of each protein is indicated. Protein levels are expressed as optical density of the bands in arbitrary units (a.u.). Values are mean ± SD of three independent experiments (*n* = 3 technical replicates for each experimental point) performed with three different cell lines of adult human skin telocytes and fibroblasts. ### *p* < 0.001 vs. untreated, *** *p* < 0.001 vs. TGFβ1 (Tukey’s test).

As illustrated in Figure 6, these results were also successively confirmed at the protein level with Western blotting, which indeed showed, in TGFβ1-alone-stimulated skin fibroblasts, a significant increase in both α-SMA and COL1A1, as well as in the myofibroblast markers N-cadherin and fibronectin containing the alternatively spliced extra domain A (FN-EDA). All these TGFβ1-induced protein level upregulations were significantly prevented when skin fibroblasts were administered with TGFβ1 in combination with TC-CM (Figure 6).

**Figure 7:**
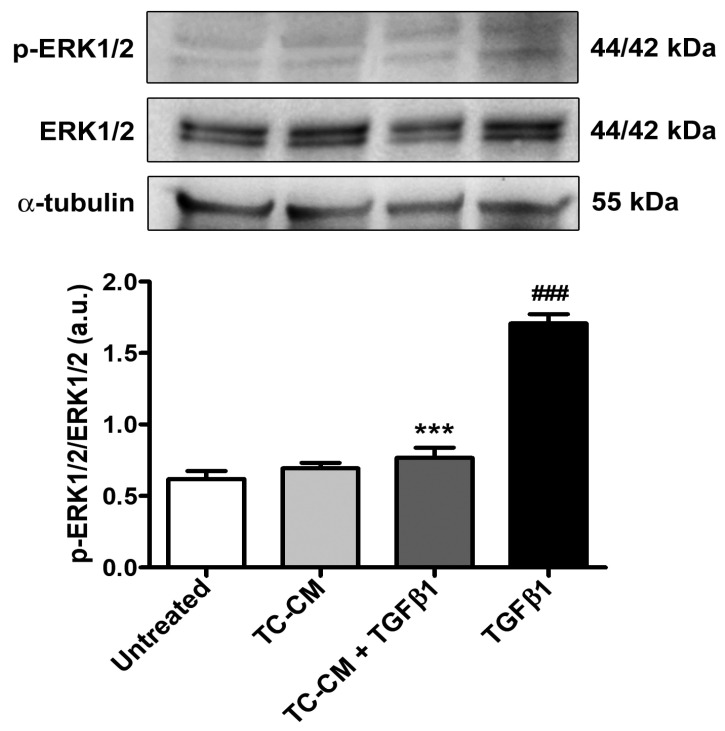
Skin telocyte conditioned medium (TC-CM) significantly prevents the transforming growth factor β1 (TGFβ1)-induced activation of extracellular-signal-regulated kinase 1/2 (ERK1/2) signaling in skin fibroblasts. Demonstrative bands of immunoblots for phosphorylated-ERK1/2 (p-ERK1/2), total ERK1/2, and α-tubulin (loading control). The molecular weight (kDa) of each protein is indicated. Protein levels are expressed as optical density of the bands in arbitrary units (a.u.). Values are mean ± SD of three independent experiments (*n* = 3 technical replicates for each experimental point) performed with three different cell lines of adult human skin telocytes and fibroblasts. ### *p* < 0.001 vs. untreated, *** *p* < 0.001 vs. TGFβ1 (Tukey’s test).

Next, we assessed, in each experimental condition, the protein ratio between phosphorylated-extracellular-signal-regulated kinase 1/2 (ERK1/2) and total ERK1/2 as a measure of ERK1/2 phosphorylation, which is implicated in the profibrotic TGFβ1-mediated intracellular signaling. As illustrated in Figure 7, treatment of skin fibroblast with TGFβ1 alone significantly augmented ERK1/2 phosphorylation, while TC-CM was able to strongly lessen such an effect.

**Figure 8:**
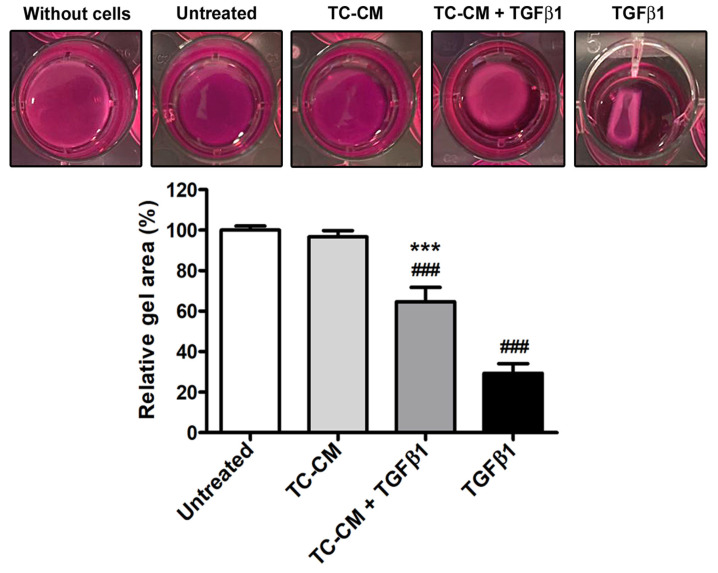
Skin telocyte conditioned medium (TC-CM) significantly dampens the transforming growth factor β1 (TGFβ1)-induced acquisition of myofibroblast contractile features by skin fibroblasts. Demonstrative collagen gel matrix contraction assay wells. Collagen gel area is presented as the percentage of the one measured for untreated fibroblasts. The experimental point without cells serves as negative control. Values are mean ± SD of three independent experiments (*n* = 3 technical replicates for each experimental point) performed with three different cell lines of adult human skin telocytes and fibroblasts. ### *p* < 0.001 vs. untreated, *** *p* < 0.001 vs. TGFβ1 (Tukey’s test).

Finally, in the skin fibroblasts stimulated with TGFβ1 in combination with TC-CM, the latter was also capable to significantly decrease the TGFβ1-promoted cell ability to contract a collagen gel matrix (Figure 8).

## 3. Discussion

The current study was conducted to investigate, for the first time, the in vitro effects of skin TC secretome as conditioned medium (TC-CM) on TGFβ1-induced skin fibroblast-to-myofibroblast transition. Our data clearly demonstrate that the TC-CM is effective in reducing TGFβ1-triggered skin fibroblast proliferation, migration, and transition to myofibroblasts, which suggests that TCs may exert a protective role against profibrotic activation of fibroblasts via paracrine mechanisms, thus contributing to the maintenance of skin homeostasis.

TCs are unique stromal cells characterized by typical prolongations (telopodes) through which they create three-dimensional networks communicating with neighboring cells either by cell–cell contacts or by shedding microvesicles and secreting signaling molecules [1,2,3,4,5,6,7,13,16,17,18,19,20,21,22]. Hence, due to their particular spatial organization, their intercellular interactions and their capability to release extracellular vesicles, TCs are thought to take part in tissue homeostasis, the imbalance of which may result in different pathologic conditions affecting a variety of organs [2,4,6,15,20,23,24,25,26,27,28,29]. Indeed, increasing evidence has proved that TC abnormalities (damage or loss) are strictly associated with many fibrosis-related diseases involving the gastrointestinal tract (e.g., Crohn’s disease, ulcerative colitis, and liver fibrosis), the genital, cardiovascular, and urinary systems (e.g., endometriosis, myocardial infarction, and renal fibrosis), as well as the skin [11,23]. In this regard, a significant derangement evolving into the loss of the TC dermal network has been described in the fibrotic skin lesions of scleroderma patients, where it was found to correlate with the extent and severity of fibrosis, as well as in the mouse model of bleomycin-induced cutaneous fibrosis [35,36]. Nevertheless, the abovementioned observations arose from descriptive studies and left open the question on whether TCs could be active contributors or simple bystanders in the complex scenario of skin fibrosis, which prompted us to undertake the current in vitro investigation.

Since, at present, there are no commercially available TC lineages, and considering that protocols isolating TCs through differentiated adhesion to cell culture plastics do not guarantee the complete separation of these cells from fibroblasts, in the present study, we used primary human skin TC lineages previously established by a two-step immunomagnetic microbead-based cell separation (i.e., negative selection for CD31 followed by positive selection for CD34) [8]. Such a methodology allowed the discrimination of these cells based on their different immunophenotypic features (i.e., CD31−/CD34+ TCs vs. CD31−/CD34− fibroblasts and CD31+/CD34+ endothelial cells), and the obtainment of primary cultures of stromal cells that effectively displayed the TC-characteristic morphological traits [8]. Moreover, by taking advantage of our immunomagnetic separation protocol, we could better recreate in vitro a human skin stromal microenvironment consisting of fibroblasts exposed to the secretome of TCs isolated from the same donor. In addition, considering that cell secretome may be affected by multiple passaging in culture, for our in vitro studies, we used TC-CM from TCs between the first and the third passages.

The outcomes of our study demonstrate the capability of skin TC-CM to significantly reduce TGFβ1-promoted expression of myofibroblast-associated markers such as α-SMA, COL1A1, and FN-EDA in skin fibroblasts. These data are in line with those recently reported by Chen et al., who indeed showed that both TC-CM and TC-derived exosomes strongly lowered the expression of the same fibrotic markers in the intrauterine adhesion (IUA) cellular model consisting of TGFβ-treated mouse endometrial stromal cells [38]. Of note, the in vitro findings of Chen et al. were also confirmed in the IUA mouse model, in which a decrease in endometrial fibrosis was observed after TC-derived exosome treatment [38]. Interestingly, a significant antifibrotic effect of TC-derived exosomes was also reported in a rat model of myocardial infarction, where direct cardiac injection of TC exosomes decreased myocardial fibrosis and improved cardiac function [20]. In another study exploring the antifibrotic effect of TCs in the bronchiolitis obliterans syndrome in vitro model, Zhang et al. demonstrated that co-culturing TGFβ-treated rat tracheal epithelial cells with pulmonary TCs was effective in diminishing the extent of epithelial-to-mesenchymal transition, suggesting a possible protective role of TCs against lung tissue fibrosis [39]. The therapeutic value of TCs was also evidenced by different studies highlighting that TC transplantation succeeded in improving fibrosis both in a rat model of myocardial infarction, where it led to a substantial reduction in infarct size and collagen deposition, and in a unilateral ureteral obstruction-induced renal fibrosis rat model, where it not only reduced fibronectin, COL1A1 and α-SMA expression, but also decreased serum TGFβ1 levels and suppressed Smad2/3 phosphorylation [40,41]. Moreover, the stimulation of the lymphatic system with specific manual techniques was reported to clinically reverse fibrosis by significantly raising the percentage of TCs in the dermis of patients affected by lymphedema, suggesting that increasing the amount of skin TCs by transplantation or by promoting their survival and growth may represent an antifibrotic therapeutic strategy [42].

As revealed by NanoSight nanoparticle tracking analysis, we found that skin TC-CM contained several extracellular vesicles with different dimensions, confirming previous research reporting that these stromal cells can release exosomes, ectosomes, and multivesicular cargos [12,13,20,38,43,44,45]. In particular, based on the mode and D90 values, we assumed that the majority of extracellular vesicles released by skin TCs in culture belonged to the category of “classical” microvesicles, i.e., small to large ectosomes ranging from 150 to 1000 nm [37]. Nevertheless, we are aware that additional investigations will be required to better understand whether the antifibrotic effect we found might be mainly ascribed to soluble factors directly released by skin TCs in the culture medium or to the extracellular vesicles, whose content should be characterized. In fact, a growing body of evidence indicates that TC-derived extracellular vesicles carry and transfer bioactive molecules such as miRNAs, non-coding RNAs, cytokines, growth factors, chemokines, glycoproteins, matrix metalloproteinases, and integrins [13,20,38,39,44,46]. In particular, in a study by Albulescu et al., mouse cardiac TC secretome containing high levels of interleukin 6, vascular endothelial growth factor, macrophage inflammatory protein (MIP)-1α, MIP-2, and monocyte chemoattractant protein-1 was shown to promote cardiac stem cell proliferation and differentiation [47]. In another study, Yang and coworkers found that mouse cardiac TC-derived extracellular vesicles carried miR30b, which was effective in reducing aortic valve calcification and valve interstitial cell apoptosis by inhibiting Wnt/β-catenin signaling pathway [48]. Furthermore, rat cardiac TC exosomes were reported to contain miRNA-21-5p, which was identified as a key functional molecule able to reduce infarct size and fibrosis in a rat model of left anterior descending coronary artery ligation-mediated myocardial infarction [49]. Finally, two very recent studies demonstrated that TC-derived exosomes also provide an important source of Wnt ligands that were shown to modulate endometrial fibrosis by acting on Wnt/β-catenin signaling pathway [38,50].

Regarding the putative molecular pathways underlying the TC antifibrotic performance, here, we demonstrated that skin TC-CM administration to skin fibroblasts was able to significantly lessen ERK1/2 phosphorylation, which is part of the profibrotic non-canonical TGFβ1 signaling. However, since it does not exclude the implication of other signaling pathways, additional studies relying on large-scale analysis could lead to a greater understanding of TC molecular mechanisms of action.

In conclusion, the present data contribute to support the protective effects of skin TCs against skin fibroblast-to-myofibroblast transition, and provide the essential background for further investigations on the feasibility of TC secretome administration or TC transplantation as innovative therapeutic approaches to fight cutaneous fibrosis. As a matter of priority, it would be of great interest to further assess whether skin TC-CM may exert a protective effect on cutaneous fibrosis even when directly injected in vivo in the bleomycin-induced skin fibrosis mouse model.

## 4. Materials and Methods

### 4.1. Culture of Primary Human Skin Telocytes and Fibroblasts

As described in detail elsewhere [8], skin plastic surgery remnants (*n* = 3 donors) underwent a two-step immunomagnetic microbead-based cell isolation process, from which three types of primary human cell lines were obtained: (i) CD31−/CD34+ TCs, (ii) CD31+/CD34+ endothelial cells, and (iii) CD31−/CD34− fibroblasts. Successively, CD31−/CD34+ TCs and CD31−/CD34− fibroblasts (*n* = 3 cell lines, each) were mantained in complete growth medium composed of 4.5 g/L glucose Dulbecco’s Modified Eagle Medium (DMEM; 11-965-092; Thermo Fisher Scientific, Waltham, MA, USA), 1% L-Glutamine (ECB3000D; Euroclone, Milan, Italy), 1% penicillin/streptomycin (ECB3001D; Euroclone), and 10% extracellular vesicle-depleted fetal bovine serum (FBS; S181M; Biowest, Nuaillé, France) and kept at 37 °C in a CO_2_ incubator. When confluent, cells were detached and pelleted or plated on different supports, depending on the experiment to perform. TCs were used between the 1st and the 3rd passages, while fibroblasts between the 3rd and 7th passages.

### 4.2. Collection of Human Skin Telocyte Conditioned Medium

For the preparation of TC-CM, confluent skin TCs were washed twice with phosphate-buffered saline (PBS) and serum-starved overnight. The culture supernatant was then collected and subjected to centrifugation firstly at 300× *g* for 5 min to discard the cell pellet, and then at 3000× *g* for half an hour at 4 °C to remove cell debris and apoptotic bodies. TC-CM was immediately used for in vitro assays in order to prevent freezing-induced extracellular vesicle destruction.

### 4.3. Extracellular Vesicle Measurements in the Conditioned Medium of Human Skin Telocytes

To measure extracellular vesicles in TC-CM, we performed a nanoparticle tracking analysis with a NanoSight NS300 instrument (Malvern Panalytical, Westborough, MA, USA) furnished with a 488 nm excitation laser and an automated syringe sampler. NanoSight technology estimates particle size on the basis of the relationship between their Brownian motion and hydrodynamic diameter through the Stokes–Einstein equation. For each sample, diluted 1:500 in PBS and loaded into 1 mL syringes, five independent records were collected. CSV files generated by the nanoparticle tracking analysis software v3.2 were used for the computational analysis.

### 4.4. Fibroblast Stimulation

Before each experiment, each fibroblast line was starved for 2 h in basal medium containing 2% extracellular vesicle-depleted FBS, and then challenged with 10 ng/mL of recombinant human TGFβ1 (PeproTech, Rocky Hill, NJ, USA) to induce fibroblast-to-myofibroblast transition or with the conditioned medium of TCs isolated from the same skin tissue donor, alone or with TGFβ1. After 48 h stimulation, cells were assessed for viability, proliferation, and gene expression, while protein expression and contractile abilities were evaluated after 72 h.

### 4.5. Cell Viability Assessment by Flow Cytometry

Skin fibroblasts, cultured into 6-well plates until 90% confluence and challenged for 48 h as described above, were collected with Accutase (ECB3056D; Euroclone) and subsequently used for the annexin V/PI flow cytometer assay with a BD FACS Canto II flow cytometer (BD Biosciences, Franklin Lakes, NJ, USA) [51]. The proportion of viable, early apoptotic, late apoptotic, and necrotic cells was calculated on the basis of the different annexin V and/or PI positivity. For each sample, tested in triplicate, no less than 10,000 events were collected.

### 4.6. WST-1 Proliferation Assay

Skin fibroblasts, cultured into 96-well plates (9 × 10^3^ cells per well) and stimulated for 48 h as described above, were subjected to the WST-1 assay (5015944001; Roche, Basilea, Switzerland) to quantify their proliferation rate. Each fibroblast line was assayed in triplicate, and data were expressed as percentage of the reduction/increase in cell proliferation over the proliferative response of untreated fibroblasts (i.e., proliferative effect with the starvation medium).

### 4.7. In Vitro Scratch Assay

Cell migration was assessed by performing the in vitro scratch assay on 6-well plates in which confluent fibroblasts were seeded in complete DMEM. After 24 h of serum-starvation, a wound was made with a 200 μL pipette tip on the cellular monolayers that, once cleaned of all detached cells by removing the medium, were treated as previously described. To assess fibroblast migratory skills, phase-contrast images of the scratched area were captured immediately after scratching and 24 h later under a Mateo TL RUO microscope (Leica Microsystems, Mannheim, Germany). Images were then compared to quantify the wound closure rate. For each cell line, three technical replicates of all experimental points were performed.

### 4.8. Cell Morphology Evaluation

To highlight TC morphological features and visualize their typical cellular shape, wheat germ agglutinin (WGA) fluorescent staining was performed. Indeed, WGA conjugates are commonly employed to label the plasma membrane thanks to their ability to bind the cellular glycocalyx. Briefly, paraformaldehyde-fixed TCs at the second passage were firstly rinsed in PBS and then incubated at room temperature for 10 min in the dark with Alexa Fluor-488-conjugated WGA (W11261; Thermo Fisher Scientific) at 1:100 dilution. Nuclei were counterstained with 4′,6-diamidino-2-phenylindole (DAPI), and images were captured with a Leica DM4000-B microscope furnished with a Leica DFC310 FX 1.4-megapixel digital color camera and the Leica software application suite LAS V3.8 (Leica Microsystems). Variations in fibroblast morphology were observed by acquiring phase-contrast pictures under a Mateo TL RUO inverted microscope (Leica Microsystems).

### 4.9. Fluorescence Immunostaining

Fibroblasts were left to grow on 20 × 20 glass coverslips, 72 h-stimulated as described above, and chemically fixed with 3.7% buffered paraformaldehyde. Cell membranes were permeabilized with 0.1% Triton X-100 in PBS for 10 min, while nonspecific binding sites were blocked with 1% bovine serum albumin in PBS for 1 h at 20 °C. Cells were then incubated overnight at 4 °C with mouse monoclonal anti-α-SMA (1:100; ab7817; Abcam, Cambridge, UK) and rabbit monoclonal anti-COL1A1 (1:300; #39952; Cell Signaling Technology, Danvers, MA, USA) primary antibodies. Incubation with irrelevant isotype- and concentration-paired IgG (Sigma-Aldrich, St. Louis, MO, USA) was performed to obtain negative controls. After 24 h, cells were covered for 45 min with Alexa Fluor-488-conjugated and Rhodamine Red-X-conjugated IgG (1:200; Invitrogen, Carlsbad, CA, USA) secondary antibodies, and then, nuclei were counterstained in blue for 10 min with DAPI, always working in the dark and at 20 °C. Immunolabeled fibroblasts were photographed using a Leica DM4000-B microscope furnished with a Leica DFC310 FX 1.4-megapixel digital color camera and the Leica software application suite LAS V3.8 (Leica Microsystems).

### 4.10. Gene Expression Assessment by Quantitative Real-Time PCR

Total RNA, extracted from fibroblasts after 48 h of treatment by means of the RNeasy Micro Kit (74004; Qiagen, Milan, Italy), was firstly quantified with a NanoDrop 8000 spectrophotometer (Thermo Fisher Scientific), and successively reverse-transcribed to cDNA that was then employed in SYBR Green real-time PCR experiments [52]. A list of the employed oligonucleotide primer pairs (QuantiTect primer assays; Qiagen) is shown in Table 1. 18S ribosomal RNA (*RRN18S*) was used as housekeeping gene to normalize data. In order to determine differences in gene expression and relative quantification, threshold cycle (Ct) and comparative Ct methodology were employed, respectively. All experimental conditions were carried out with three technical replicates for each of the three human skin fibroblast lines.

### 4.11. Protein Expression Assessment by Western Blotting

The 72 h-treated fibroblasts were collected in order to obtain cellular pellets, from which proteins were extracted by lysing cells with Ripa buffer (89901; Thermo Fisher Scientific) additioned with a cocktail of protease inhibitors (11697498001; Roche), NaF and sodium orthovanadate, both 1 mM. After sonication of lysates, the amount of proteins was determined by means of Bradford’s protein assay. For each sample, added with Laemmli sample buffer (Bio-Rad, Hercules, CA, USA) and β-mercaptoethanol, and subsequently boiled for 5 min at 90 °C, 30 µg of proteins was subjected to the electrophoretic run and transferred onto a nitrocellulose membrane by using the Trans-Blot Turbo Mini 0.2 µm Nitrocellulose Transfer Packs (#1704158; Bio-Rad). The employed primary antibodies are listed in Table 2. Protein bands were revealed with the ChemiDoc Touch Imaging System (Bio-Rad), and each band densitometry was performed using ImageJ software 64-bit Java 1.8.0_112 Windows version (NIH, Bethesda, MD, USA; online at http://rsbweb.nih.gov/ij, accessed on 15 February 2024).

### 4.12. Collagen Gel Matrix Contraction Assay

By following the instructions of the commercial floating matrix model kit (CBA-5020; Cell Biolabs, San Diego, CA, USA), 72 h-challenged fibroblasts were collected and resuspended in DMEM containing 2% extracellular vesicle-depleted FBS (2 × 10^6^ cells/mL). An amount of 500 µL of a solution made of 100 µL of cell suspension and 400 µL of collagen gel matrix solution was then added to each well of the kit-supplied adhesion-resistant-matrix-coated 24-well plate. Negative controls were obtained by using gels without the addition of the cell suspension. After placing the 24-well plate in a CO_2_ incubator for 1 h in order to let the collagen gel matrix polymerize, each gel matrix was covered with basal medium or medium supplemented with the different stimuli. Three technical replicates of all experimental conditions were performed. Photographs of the plates were captured after 24 h, and the area of each gel was quantified with ImageJ software 64-bit Java 1.8.0_112 Windows version (NIH; online at http://rsbweb.nih.gov/ij, accessed on 4 March 2024).

### 4.13. Statistical Data Analysis

All statistical analyses were carried out with GraphPad Prism 5 software. In particular, once data normality was confirmed with Kolmogorov–Smirnov and Shapiro–Wilk tests, the comparison of the three groups was performed by using one-way analysis of variance (ANOVA) followed by post hoc Tukey’s test. Data were stated as mean ± standard deviation (SD). In case of *p* < 0.05, differences were considered statistically significant.

## Figures and Tables

**Table 1:** Oligonucleotide primer pairs employed for quantitative PCR.

Gene	Assay ID	Catalog Number
*FAP*	Hs_FAP_1_SG	QT00074963
*ACTA2*	Hs_ACTA2_1_SG	QT00088102
*COL1A1*	Hs_COL1A1_1_SG	QT00037793
*COL1A2*	Hs_COL1A2_1_SG	QT00072058
*FN1*	Hs_FN1_1_SG	QT00038024
*CTGF*	Hs_CTGF_1_SG	QT00052899
*RRN18S*	Hs_RRN18S_1_SG	QT00199367

**Table 2:** Western blotting primary antibodies.

Primary Antibody	Catalog Number	Producer	Dilution
anti-α-SMA	ab7817	Abcam	1:300
anti-N-cadherin	#13116S	Cell Signaling Technology	1:1000
anti-COL1A1	#39952	Cell Signaling Technology	1:1000
anti-FN-EDA	SAB4200880	Sigma-Aldrich	1:1000
anti-ERK1/2	ab17942	Abcam	1:1000
anti-p-ERK1/2	sc-16982	Santa Cruz Biotechnology	1:1000
anti-GAPDH	ab8245	Abcam	1:5000
anti-α-tubulin	#2144	Cell Signaling Technology	1:1000

α-SMA—α-smooth muscle actin; COL1A1—α-1 chain of type I collagen; ERK1/2—extracellular signal-regulated kinase 1/2; FN-EDA—fibronectin containing the alternatively spliced extra domain A; GAPDH—glyceraldehyde 3-phosphate dehydrogenase; p-ERK1/2—phosphorylated-ERK1/2.

## Data Availability

The original contributions presented in the study are included in the article, further inquiries can be directed to the corresponding author.
